# Threshold voltage instability and polyimide charging effects of LTPS TFTs for flexible displays

**DOI:** 10.1038/s41598-021-87950-0

**Published:** 2021-04-16

**Authors:** Hyojung Kim, Jongwoo Park, Taeyoung Khim, Sora Bak, Jangkun Song, Byoungdeog Choi

**Affiliations:** 1 Samsung Display Co., Ltd, Technology Quality Reliability in OLED Business, Asan-si, 31454 Korea; 2 Department of Semiconductor and Display Engineering, Sungkyunkwan University, Suwon-si, 16419 Korea; 3 Department of Electrical and Computer Engineering, Sungkyunkwan University, Suwon-si, 16419 Korea

**Keywords:** Electrical and electronic engineering, Nanoscience and technology, Nanoscale devices, Techniques and instrumentation, Electronics, photonics and device physics

## Abstract

In this paper, we investigate the V_th_ shift of p-type LTPS TFTs fabricated on a polyimide (PI) and glass substrate considering charging phenomena. The V_th_ of the LTPS TFTs with a PI substrate positively shift after a bias temperature stress test. However, the V_th_ with a glass substrate rarely changed even with increasing stress. Such a positive V_th_ shift results from the negative charging of fluorine stemmed from the PI under the gate bias. In fact, the C–V characterization on the metal–insulator-metal capacitor reveals that charging at the SiO_2_/PI interface depends on the applied gate bias and the PI material, which agrees well with the TCAD simulation and SIMS analyses. As a result, the charging at the SiO_2_/PI interface contributes to the V_th_ shift of the LTPS TFTs leading to image sticking.

## Introduction

Recently, flexible displays have attracted considerable attention as next-generation drivers in display industries^1,2^. Because low-temperature polycrystalline silicon (LTPS) thin-film transistors (TFT) have the advantages of low temperature process integration and high mobility, they have rapidly become popular in flexible displays and applications. However, the usage of excimer laser annealing (ELA) for the crystallization of amorphous silicon (a-Si) and chemical vapor deposition (CVD) for the process of gate insulation produce hysteresis loops in the device characterization, due mainly to the material and physical constraints of defects and non-uniformity^3,4^. In turn, such intrinsic limitations impact fluctuations of electric current that flows into the organic light emitting diodes (OLED), leading to TFT instability and display performance disturbance, including halo and image sticking^5,6^. In addition, moisture and oxygen penetration are serious deficiencies of all plastic substrates available for OLED flexible display applications^7,8^. In particular, OLED's lifetime decreases when exposed to oxygen and moisture. When fabricating a device on a flexible plastic substate, an inorganic layer (barrier) such as SiO_2_, SiN_x_, or Al_2_O_3_ is deposited on the plastic substrate to prevent moisture and oxygen from penetrating into the OLED^9^. Although the main causes of residual images on OLED displays are inherently related to the process variation of the backplane process and deterioration of the OLED material^10,11^, TFT instability can aggravate image sticking under low frequencies and always-on-display applications^12^. A few papers are available for the elucidation of the mechanism and reliability physics of the positive V_th_ shift of p-type LTPS TFT and image sticking in regards to the substrate material, process integrity, and bias stress test conditions^13^.

In this paper, information is given to elucidate the mechanism of the positive V_th_ shift when the gate bias is applied to p-type LTPS TFTs fabricated on PI, as compared to a glass substrate. In order to probe the effects of charging on LTPS TFTs, three different metal-insulation-metal (MIM) capacitors were prepared for the C–V characterization. Finally, we delve into V_th_ shift behaviors in the material properties of PI associated with the vertical structure of LTPS TFTs. The secondary ion mass spectrometry (SIMS) physical characterization and TCAD simulation are focused on the fluorine profile at the interface and effects of the polarity of charging on V_th_ shift behaviors.

## Results and Discussion

### V_th_ shift behaviors of LTPS TFTs fabricated on PI and glass substrates

**Figure 1:**
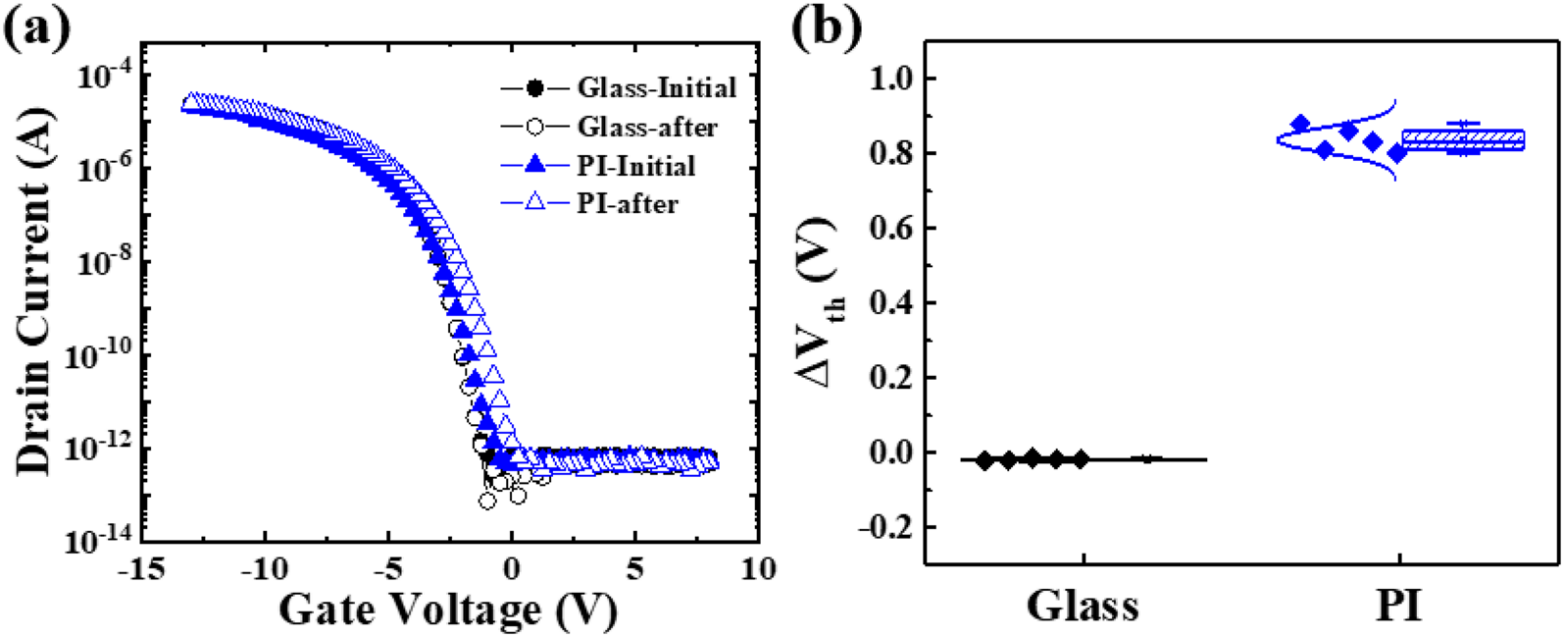
(**a**) I_D_–V_G_ plot of P-type LTPS TFTs before and after the BTS. Note that the P-type LTPS TFTs were fabricated on glass (black filled circle) and PI (blue filled triangle) substrates and (**b**) ΔV_th_ before/after BTS of TFTs fabricated on glass and PI substrates.

Figure 1a shows the I_D_–V_G_ plot of p-type LTPS TFTs with glass and PI substrates on the top gate structure, before and after a gate stress of − 30 V for 4,000 s at 70 °C. In Fig. 1b, ΔV_th_ after BTS was plotted as a box plot using 5 TFTs fabricated on the glass and PI substrate. On average, the ΔV_th_ of the TFT fabricated on glass showed a slight change, less than − 0.02 V, and the ΔV_th_ of the TFT fabricated on the PI showed a positive shift with an average 0.83 V. In addition, I_on_ increased from 2.08 × 10^–5^ A to 2.46 × 10^–5^ A, and field effect mobility increased from 85 to 113 cm^−2^. However, under the identical stress conditions, the parameters of the LTPS TFT fabricated on glass substrate are rarely changed. It is somewhat interesting to note that the V_th_ of p-type LTPS TFTs with a PI substrate is positively shifted under the negative gate bias. It is speculated that the negative charging is generated from the PI substrate below the gate insulation layer.

**Figure 2:**
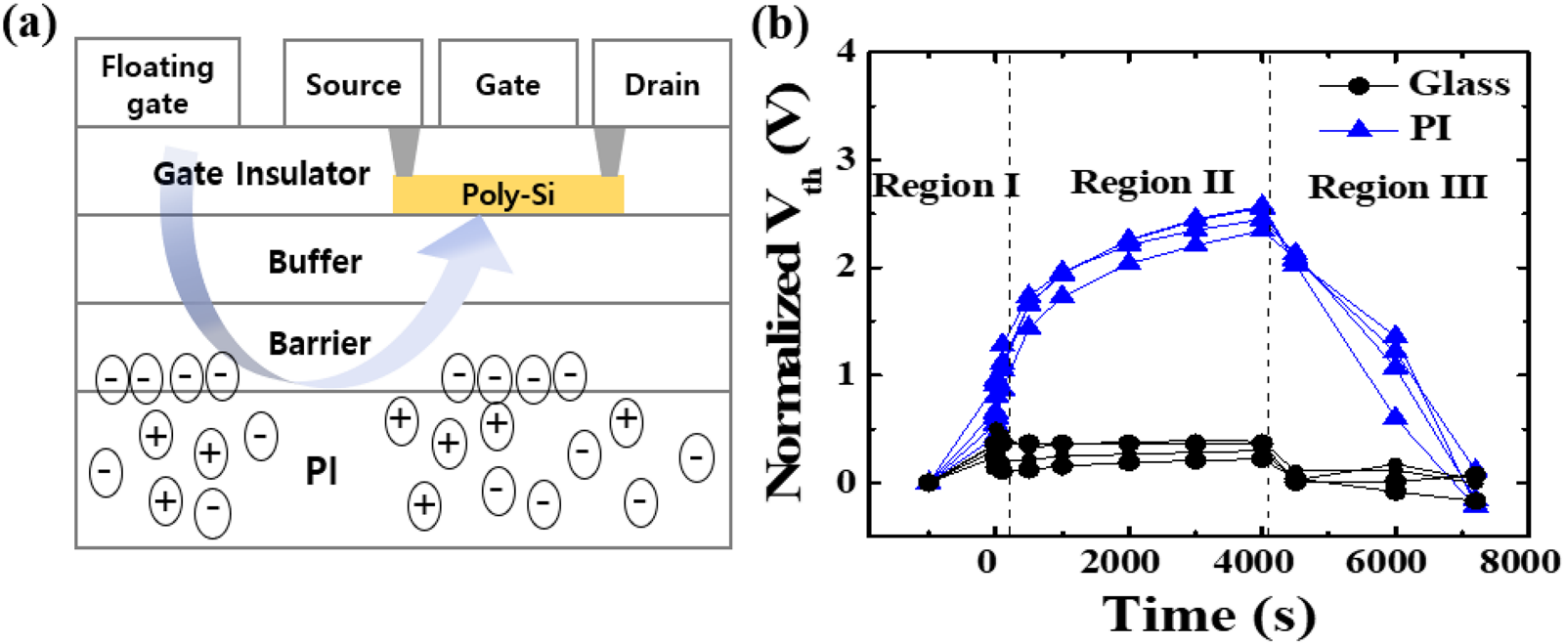
(**a**) Schematic of 4-pad characterization (floating gate, source, gate and drain) and (**b**) normalized V_th_ behaviors of P-type LTPS TFTs fabricated on the on glass (black filled circle) and PI (blue filled triangle) substrates.

In order to further probe the positive V_th_ shifts, 4-pad evaluation was conducted. As shown in Fig. 2a, a bias of 50 V was applied to the floating gate adjacent to the LTPS TFT to minimize the effects of the poly channel on electrical charging in the PI substrate. V_th_ behaviors are shown in Fig. 2b. As shown, the LTPS TFT with a glass substrate demonstrates stabilized V_th_ behaviors throughout stressing at 70 °C up to 80,000 s. Note that Region I, II, and III represent the temperature and bias conditions used; 70 °C without bias, 70 °C with 50 V, and room temperature without bias, respectively. From Region I to II, the V_th_ of the TFT with a PI substrate increases and positively shifts with increasing time. The V_th_ tends to decrease and then return to the initial V_th_ after 1 h in Region III. In turn, Region III is intended to observe the recovery behaviors of the V_th_ of the LTPS TFT with a PI substrate. Since the recovery of the V_th_ observed in Region III could result in display image disturbance, such as image sticking defined as residual images, efforts in electrical and physical characterization are inevitable. One would argue that the positive V_th_ shifts of the TFT with a PI substrate mentioned are related to design flaws somehow associated with the layout design of the metal route, which can impact TFT stability as electrical fields arise from adjacent metals near TFT devices. However, such artifacts were eliminated by design review throughout circuit simulation.

**Figure 3:**
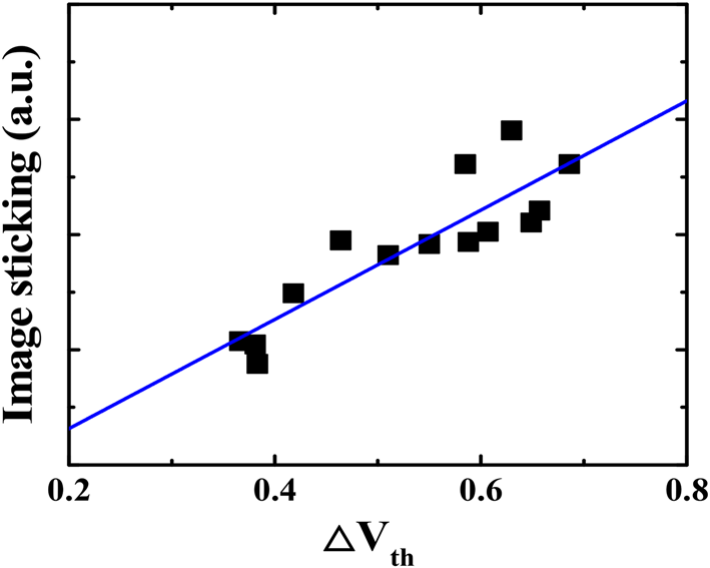
Correlation between PI charging induced V_th_ shift and image sticking for an LTPS TFT with a PI substrate.

Since the V_th_ stability of TFT devices plays an important role for display image performance, particularly for image sticking, the relationship between the ΔV_th_ vs. image sticking was investigated. Using the checkerboard test pattern, the image sticking index is estimated by comparing luminance changes before and after stressing^13^. The white area adjacent to black patterns in the checkerboard becomes darker after stressing, which indicates image sticking. Figure 3 shows the correlation between PI charging induced ΔV_th_ and image sticking. It is shown that the larger the ΔV_th,_ the higher the propensity for image sticking. These results show that the correlation between V_th_ shift and image sticking needs to be further discussed with electrical and physical characterization.

### MIM capacitor fabrication, C–V measurement and physical SIMS characterization

**Figure 4:**
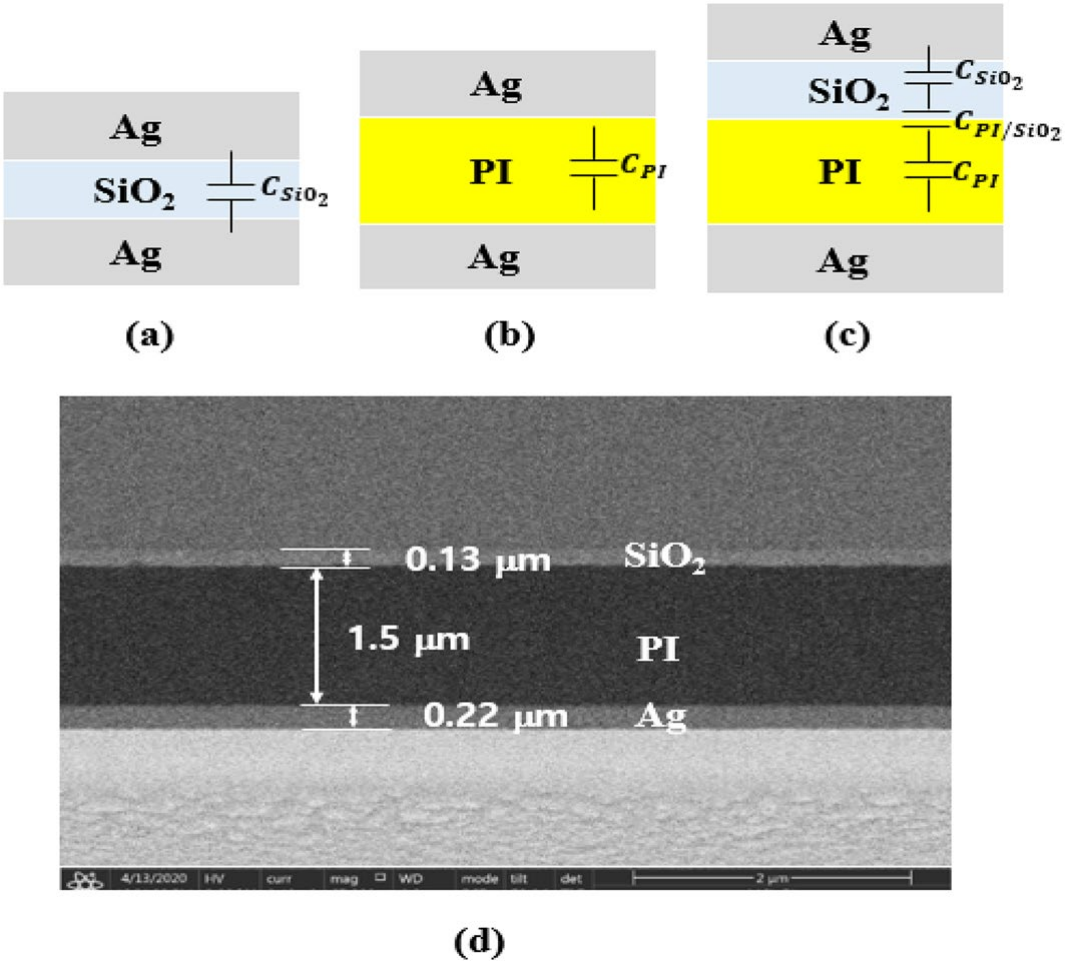
Schematics of the vertical structure for the MIM capacitors and SEM image; (**a**) Ag/SiO_2_/Ag, (**b**) Ag/PI/Ag, (**c**) Ag/SiO_2_/PI/Ag and (**d**) the cross-sectional analysis of the MIM capacitor shown in (**c**).

In an effort of probing the V_th_ behaviors mentioned above, three different metal–insulator-metal (MIM) capacitors, such as Ag/PI/Ag, Ag/SiO_2_/Ag and Ag/SiO_2_/PI/Ag were prepared for the C–V measurements conducted by varying voltage at a frequency of 100 kHz. SIO_2_ is the barrier layer of the LTPS TFT, and the thickness and process conditions are the same with the measured TFT. Figures 4a–d show the schematics of the vertical structure for the MIM capacitors, i.e., Ag/SiO_2_/Ag, Ag/PI/Ag, Ag/SiO_2_/PI/Ag, and the cross-sectional analysis of the MIM capacitor. An Ag metal electrode was sputtered onto the spin-coated PI and SiO_2_, deposited using the PECVD process. As a result, the PI and PI/SiO_2_ function as the insulators between the metal electrodes.

Using Eq. (1), the physical dimension, dielectric constant, and capacitance of each capacitor are estimated and summarized in Table 1.

1 $$\frac{{1}}{{{\text{C}}_{{{\text{Total}}}} }}{ = }\frac{{1}}{{{\text{C}}_{{{\text{SiO}}_{{2}} }} }}{ + }\frac{{1}}{{{\text{C}}_{{{\text{PI}}}} }}{ + }\frac{{1}}{{{\text{C}}_{{{\text{PI/SiO}}_{{2}} }} }}$$

**Table 1:** MIM capacitance and parameters from C–V measurement.

	SiO_2_	PI	PI/SiO_2_
Thickness (um)	0.13	1.5	–
Dielectric constant	3.8	3.91	–
Capacitance (F)	6.37 × 10^–9^	7.56 × 10^–10^	9.39 × 10^–11^
ΔQ/q for 30 V stress (/cm^2^)	2.7 × 10^10^	5.3 × 10^10^	2.0 × 10^11^

**Figure 5:**
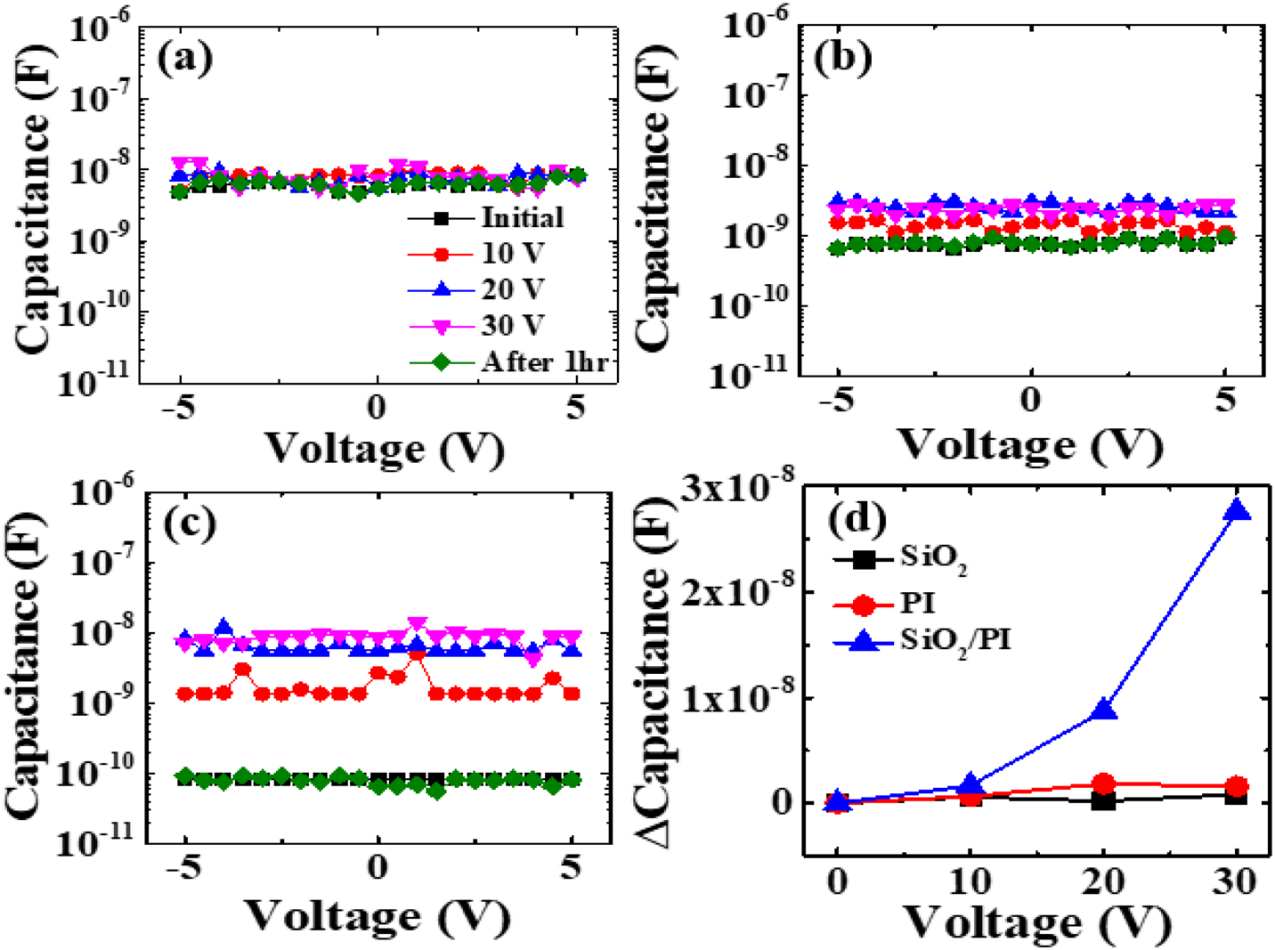
C–V characterization for 3 different MIM capacitors with increasing voltage; (**a**) Ag/SiO_2_/Ag, (**b**) Ag/PI/Ag, (**c**) Ag/SiO_2_/PI/Ag, and (**d**) the dependency of the MIM capacitors on the applied voltage.

Figure 5 shows the results obtained from the C–V measurements. It is apparent that changes in capacitance depend on the MIM capacitors. The capacitances of the SiO_2_ and PI dielectric insulators between the Ag electrodes rarely change, even with increasing voltage (see Fig. 5a,b). However, the capacitance of the SiO_2_/PI dielectric tends to increase with increasing voltage. It was also found that the magnitude of capacitance turns into the initial state after 1 h of halting bias, as shown in Fig. 5c. In Fig. 5d, the capacitance of the SiO_2_/PI rapidly increases with increasing voltage. Such results agree well with the recovery of the V_th_ described in Fig. 2b. Thus, it is legitimate that the positive V_th_ shift of a TFT with a PI substrate is attributed to charging between the SiO_2_ and PI interface. As such, charge generation at the interface between the SiO_2_ and the PI plays an important role for TFT device stability, particularly for the PI substrate.

**Figure 6:**
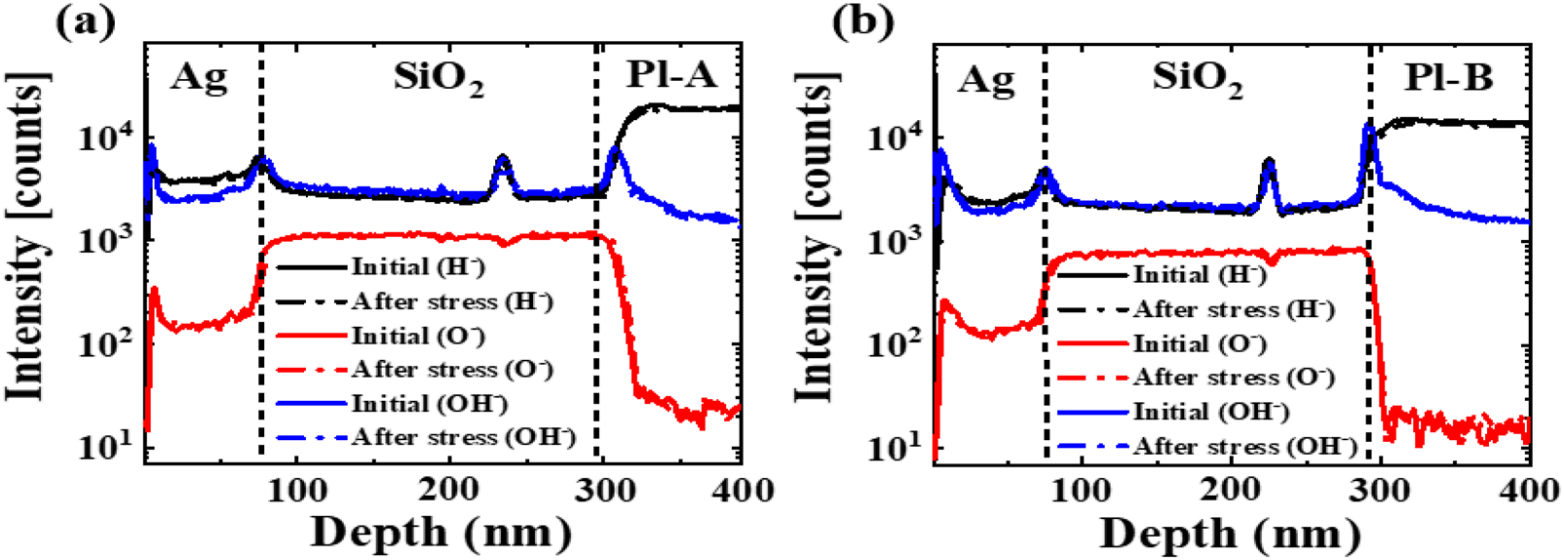
Hydrogen, hydroxy group and oxygen profiles obtained by SIMS measurements of capacitors before/after bias stress; (**a**) Ag/SiO_2_/PI-A/Ag and (**b**) Ag/SiO_2_/PI-B/Ag.

**Figure 7:**
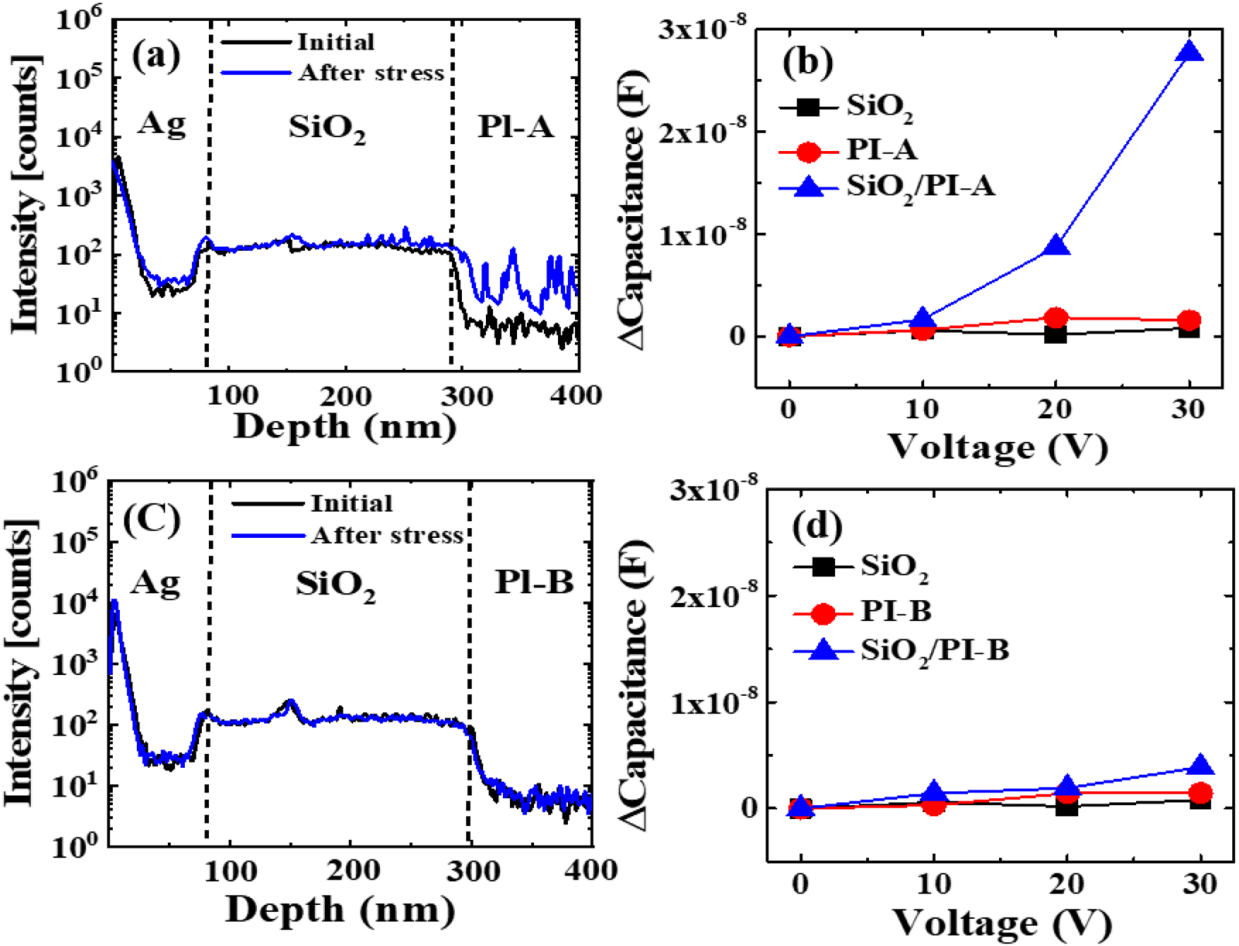
SIMS characterization of Ag/SiO_2_/PI/Ag capacitor focused on fluorine profile at the interface; (**a**, **b**) with PI-A type and (**c**, **d**) with PI-B type. Note that PI-A is less crosslink density than PI-B.

In sequence, SIMS analysis is adopted to explicate the positive V_th_ shifts of p-type LTPS TFTs with a PI substrate. To understand the effects of PI on the V_th_ shift, two different PIs with low and high crosslink density, named PI-A and PI-B, were chosen to prepare the Ag/SiO_2_/PI/Ag capacitor. We first suspected that oxygen or moisture had penetrated from the PI. Changes in hydrogen ions (H^−^), hydroxyl group (OH^−^), and oxygen ions (O^−^) before/after bias stress of Ag/SiO_2_/PI/Ag capacitors were confirmed through SIMS analysis. Figure 6 shows the results of SIMS analysis before and after bias stress of MIM capacitors fabricated based on PI-A and PI-B. There was no change in H^−^, OH^−^, or O^−^ before/after bias stress, confirming that there was no penetration of oxygen or moisture from PIs. Figure 7 shows the correlation between the fluorine profile at the SiO_2_/PI interface and capacitance, characterized by SIMS and C–V measurement. Comparatively, the SIMS analyses revealed that PI-A has a higher florin content than PI-B at the interface (see Fig. 7a,c). In consequence, C–V measurements shown in Fig. 7b,d show that the capacitance of the MIM capacitor with PI-B is rarely changed even with increased stressing at 70 °C. It has been reported that the negative fluorine ions, F^-^, are subjected to transfer and trapped in the SiO_2_ under the bias^14^. As a result, SIMS analyses evidences that a mobile ion F^-^ in the SiO_2_/PI interface contributes to the positive shifts of LTPS TFTs with a PI substrate. This suggests that the amount of charging generated in the PI is dependent on the material property of the PI. Hence, the material property of the PI is a crucial factor that can influence LTPS TFTs with a PI substrate. It has been also found that PI charging that significantly affects TFT reliability can be successfully suppressed by the selection of a proper PI with high volume resistivity^15^. However, the charge generation and transfer into the barrier layer remained in unripe areas.

### TCAD simulation for charge generation at the interface between the SiO_2_ and PI

**Figure 8:**
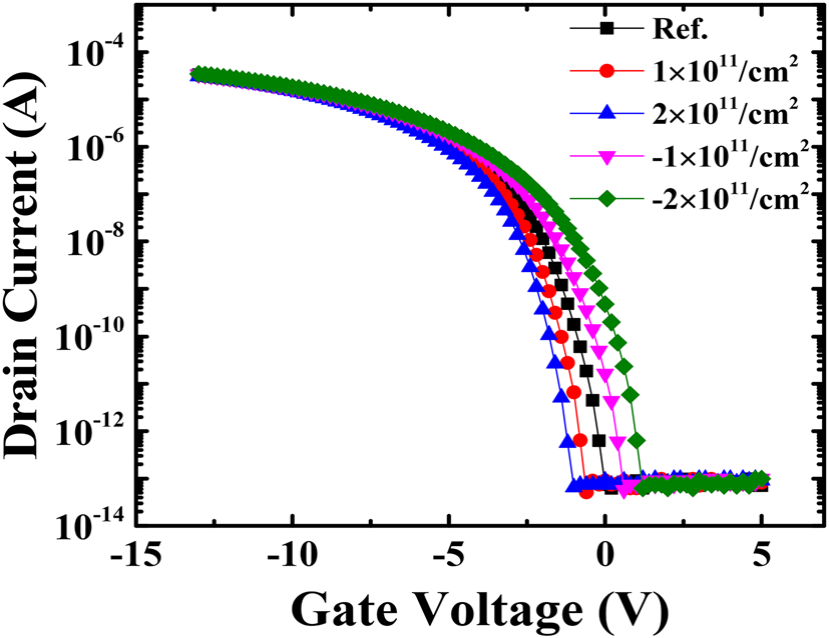
I_D_–V_G_ plot based on the amount of charge trapped at the SiO_2_ and PI interface by TCAD simulation.

Based on empirical data collected from reliability assessments, Silvaco TCAD was used to simulate the effects of charging at the SiO_2_/PI interface on the TFT transfer curve. Figure 8 shows the I_D_–V_G_ plot of an LTPS TFT with a PI substrate. As shown, the negative charging in the SiO_2_/PI interface shifts the V_th_ to the positive direction, while the positive charging results in a negative V_th_ shift. Accordingly, when − 2 × 10^11^/cm^2^ charging is generated at the interface between the SiO_2_ and the PI, the estimated V_th_ shift toward the positive direction is 0.84 V.

Table 2 contains the estimated TFT parameters, such as V_th_, μ_FE_, subthreshold swing (SS), and on/off ratio, based on the given charge injections shown in Fig. 8.

**Table 2:** Estimated TFT parameters with respect to charge injection from the SiO_2_/PI interface by TCAD simulation.

Charge Injection (/cm^2^)	V_th_ (V)	μ_FE_ (cm^2^/V-s)	SS (V/dec)	On/off ratio
Ref	− 1.47	82	0.43	4.21 × 10^8^
1 × 10^11^	− 1.92	75	0.40	3.93 × 10^8^
2 × 10^11^	− 2.27	69	0.33	3.79 × 10^8^
− 1 × 10^11^	− 1.02	90	0.45	4.57 × 10^8^
− 2 × 10^11^	− 0.63	101	0.46	5.58 × 10^8^

**Figure 9:**
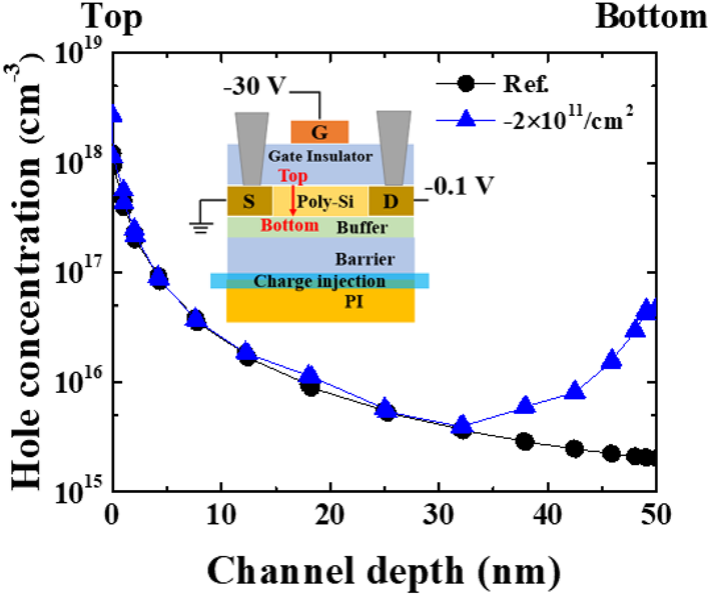
TCAD simulation for hole concentration (cm^−3^) versus channel depth (nm) of an LTPS TFT with a PI substrate. Reference represents a LTPS TFT with a PI substrate without charge injection at the SiO_2_/PI interface.

Figure 9 is given to explain the hole concentration of an LTPS TFT with a PI substrate with the bias conditioned at V_GS_ − 30 V and V_DS_ − 0.1 V. The Reference (black filled circle) and − 2 × 10^11^/cm^2^ (blue filled triangle) represent the LTPS TFT with and without charging at the SiO_2_ and PI interface. Recall that 2 × 10^11^/cm^2^ is obtained from the C-measurement summarized in Table 1. As shown in the inlet in Fig. 9, when the negative bias is applied to the gate of the LTPS TFT, hole carriers tend to be accumulated near the channel and then exponentially decrease. However, when negative charging exists at the PI substrate, hole concentration decreases then increases below the channel depth of 30 nm. It is known that the negative charges at the interface between the SiO_2_ and PI result in an early turn-on V_th_ leading to increased field effective mobility and I_on_, which is similar to the I_D_–V_G_ characteristic observed from the double gate TFTs^16,17^. Hence, the state of charging at the interface determines the characteristics of the LTPS TFTs with a PI substrate.

## Conclusions

Considering the direction of V_th_ shift of p-type LTPS TFTs with gate bias stress, the charge trapping mechanism, depending on the type of the substrate in the top gate structure, are comprehensively investigated. Unlike the glass substrate, the positive V_th_ shift of p-type LTPS TFTs with a PI substrate under the BTS test results from the negative charging of fluorine at the interface between the SIO_2_ and PI is proven by C–V measurement and SIMS characterization. In fact, the fluorine stems from the PI substrate under gate bias stress. Furthermore, TCAD simulation reveals that the direction of the V_th_ shift strongly depends on the polarity of charge trapping at the SiO_2_ and PI interface. The larger V_th_ shifts are prone to the higher propensity of image sticking. Hence, care must be taken for the selection of PI in order to ensure display image performance in advanced flexible display technologies.

## Methods

P-type LTPS TFTs were fabricated on either a PI or glass substrate through the standard backplane process, in which plasma enhanced chemical vapor deposition (PECVD) was used for a-Si and SiO_2_ (barrier) deposition and crystallized into poly-Si by using excimer laser annealing (ELA). Thus, the vertical structure of the p-type LTPS TFTs consists of the gate/gate insulator/poly-Si/buffer/barrier/PI or glass substrate on the top gate structure. In a pixel circuit driver, 4/4 μm transistor and 200 μm/200 μm capacitance were monitored by I–V and C–V measurement. V_th_ behaviors were monitored as a function of bias stress on the gates of the LTPS TFTs. To probe electrical charging in PI, three different metal–insulator-metal (MIM) capacitors, Ag/PI/Ag, Ag/SiO_2_/Ag, and Ag/SiO_2_/PI/Ag, were prepared for the C–V measurements. Changes in capacitance were measured as a function of voltage level. Moreover, the effects of charging in PI on the LTPS TFTs were taken into accounted by selecting different types of PIs such as PI-A and PI-B. Fluorine profiles were carefully analyzed by SIMS characterization. Finally, the effects of PI charging on the LTPS TFTs were consummated by using TCAD simulation.
